# Infant Nutrition and Later Health: A Review of Current Evidence

**DOI:** 10.3390/nu4080859

**Published:** 2012-07-26

**Authors:** Siân Robinson, Caroline Fall

**Affiliations:** MRC Lifecourse Epidemiology Unit, University of Southampton, Southampton General Hospital, Southampton SO16 6YD, UK; Email: chdf@mrc.soton.ac.uk

**Keywords:** infant, breastfeeding, formula feeding, weaning, programming, lifecourse

## Abstract

There is a growing recognition of the need for a lifecourse approach to understanding the aetiology of adult disease, and there is now significant evidence that links patterns of infant feeding to differences in health outcomes, both in the short and longer term. Breastfeeding is associated with lower rates of infection in infancy; in high-income populations, it is associated with reductions in blood pressure and total blood cholesterol, and lower risks of obesity and diabetes in adult life. Breastfeeding rates are suboptimal in many countries, and strategies to promote breastfeeding could therefore confer important benefits for health at a population level. However, there are particular challenges in defining nutritional exposures in infancy, including marked social gradients in initiation and duration of breastfeeding. In recent studies of low and middle-income populations of children and young adults, where the influences on infant feeding practice differ, beneficial effects of breastfeeding on blood pressure, BMI and risk of diabetes have not been confirmed, and further information is needed. Little is currently known about the long-term consequences of differences in the timing and nature of the weaning diet. Future progress will depend on new studies that provide detailed prospective data on duration and exclusivity of breastfeeding together with appropriate characterisation of the weaning diet.

## 1. Introduction

Over the past two decades a substantial body of evidence has accumulated that shows clear links between environmental influences acting in early life and adult health [1,2]. Whilst much of the focus has been on fetal life, there is growing interest in the early postnatal period, and in particular, the extent to which variations in infant nutrition affect growth, development and long-term function. Many studies have compared breast and formula feeding in infancy, and less is currently known about weaning practice and the role of qualitative differences in diet in late infancy. The focus of this review is on the evidence, principally from populations in high and middle-income countries, that links variation in infant feeding to long-term outcomes.

## 2. Infant Nutrition

Much of our current understanding of the long-term influences of variation in early nutrition has come from historical studies. Inevitably, the quality and type of records of infant feeding vary, and are often based on recalled information. Contemporary prospective studies that describe infant feeding in detail are therefore essential, although they rely on markers of long-term health assessed in childhood or early adult life. Whilst most of the existing evidence is observational, there are two important experimental trials of milk feeding. In the first of these, preterm infants were randomized to receive preterm infant formula or breast milk [3]. The second is a trial (PROBIT) in which hospitals were randomized to receive routine care or a breastfeeding promotion intervention, which increased the duration and exclusivity of breastfeeding [4]. The infants in both trials have been followed up in later childhood. Across all studies a notable feature is that there is considerable variation in the description of infant feeding and the definition of nutritional exposures. These differences impact on our ability to collate findings, and they provide challenges for the interpretation of the links between infant nutrition and adult health.

### 2.1. Breast *versus* Formula Feeding

Breast milk has a complex and variable composition, which differs over time and between women. A key characteristic is its content of a wide range of bioactive constituents, which include anti-microbial and anti-inflammatory factors, enzymes, hormones and growth factors [5]. When considering observational evidence of differences in outcomes between breastfed babies and those fed on breast milk substitutes, there are particular issues that need to be considered. Firstly, there have been marked changes in the composition of breast milk substitutes over the past century, and in older studies the composition of the substitutes used may have differed significantly between individuals and populations. Apparent differences in health outcomes that are attributed to breastfeeding may therefore capture two differences in exposure—firstly, variation in exposure to breast milk—but secondly, a lack of exposure to the breast milk substitutes used in the study populations at the time. For example in the past, diluted cow’s milk was often fed instead of breast milk, whereas today use of formula milks is routine. Formula milks continue to evolve in their composition and the nature of the differences, compared with breast milk, require careful evaluation. A second problem is the definition of duration and exclusivity of milk feeding, as mixed feeding of breast and formula milk is common, and studies differ in the way that dose effects of breast milk are described. Inconsistent definitions of variation in exposure to breast milk and breast milk substitutes may contribute to misclassification of individuals, and to inconsistency across studies that examine long-term health outcomes in relation to breastfeeding. A third issue is social gradients in patterns of infant feeding, particularly in breastfeeding, that are found in high-income countries today. For example, in the national study of infant feeding in 2010, 91% of UK women who had completed full-time education over the age of 18 years initiated breastfeeding, compared with 63% of women who left school at 16 years or younger [6]. Such marked differences in family background are likely to impact on other aspects of lifestyle, including patterns of physical activity and diet in later life, which also have implications for long-term health, and may confound associations between breastfeeding and later outcomes. The older historical cohorts that have prospective records of infant feeding are therefore particularly valuable, as social gradients in infant feeding were much less evident in the past. Important insights will also come from comparison with low and middle-income countries where influences on infant feeding practice and patterns of confounding factors differ [7,8]. 

### 2.2. Weaning Practice and Diet Quality in Infancy

In comparison with types of milk feeding in infancy, we currently know less about the importance of variations in weaning practice or whether there are long-term effects of qualitative differences in the weaning diet. The age at introduction of solid foods and the type of first solids are often documented, but the assessment of food and nutrient intake during later infancy is less common, and the process of weaning—that is the gradual transition from a milk-based diet to a diet based on solid foods—is not well described [9,10]. Partly this may be a reflection of the technical challenges encountered in assessing intake in such young children, but as dietary patterns and intakes are changing rapidly in late infancy it may be difficult to determine the key stage and exposures that require definition. An important observation is that weaning practice, including the timing of introduction to solid foods, and type of weaning diet, is linked to the pattern of milk feeding in earlier infancy. Breastfed babies are commonly introduced to solid foods later than infants who are formula-fed [11,12], and factors that relate to the duration of breastfeeding, such as maternal education, also influence the nature of the weaning diet [10,12]. It is therefore important to consider both milk feeding and variations in the weaning diet when examining the influences of infant diet and nutrition on later health.

## 3. Infant Nutrition and Health Outcomes

Variations in infant feeding have been associated with a range of health outcomes both in the short and longer-term. Lower rates of infection among breastfed babies have been recognised for many years, but in the past decade a new evidence base has been established that links variations in infant nutrition to health outcomes much later in life.

### 3.1. Infection

Breast milk has been described as the “communication vehicle” between the maternal immune system and the infant [13]. It contains a wide range of bioactive factors, including immunoglobulins, lymphocytes, neutrophils, cytokines and other anti-inflammatory compounds [5,13]. These factors influence immune status by providing protection, but also promote immune development and facilitate development of tolerance and an appropriate inflammatory response [13,14]. Protective benefits of breastfeeding in infancy, particularly in relation to gastrointestinal and respiratory infections, have been described many times [15]. The differences are most marked in the developing world, where access to clean water may be an issue; ten-fold differences in risk of mortality from diarrhoea under six months have been described between exclusively breastfed and non-breastfed infants [16]. However, even in the developed world, consistent differences in infection rates are evident. For example, data from the UK Millenium Cohort of infants suggested that 53% of hospital admissions for diarrhoea and 27% for respiratory tract infections up to 8 months could have been prevented by exclusive breastfeeding [17]. There are also marked differences in the prevalence of less severe infections in infancy according to the duration of breastfeeding [18]. The importance of breastfeeding for protection from gastrointestinal infection was confirmed among infants in the PROBIT trial, although the rates of respiratory infection did not differ between infants exclusively fed for 3 or 6 months [19]. Because the bioactive components of breast milk influence immune development, protective effects could continue beyond the period of breastfeeding. Early observations suggested that this was the case, as lower rates of infection were found in children who had been breastfed in infancy [20,21], but in more recent studies, such evidence of prolonged benefit has not been confirmed [17]. 

Much less is known about other aspects of infant feeding and infections in infancy. It is possible that the immature infant may be vulnerable to infection arising from the effects of early weaning, and early introduction of solid foods has been linked to greater risk of respiratory infection [22]. However, this has not been seen in other studies, and among infants in the Millenium Cohort, risk of hospitalisation for infection was not higher in those receiving solid foods [23].

### 3.2. Allergy and Atopy

There has been intense interest in the role of infant feeding in the aetiology of asthma, eczema and other atopic conditions, with a view to understanding whether appropriate infant feeding, particularly breastfeeding, could be protective. For high-risk children, breastfeeding is commonly recommended, although its role in atopic conditions is still not well understood. The pathogenesis of immune-mediated diseases, that include allergic conditions, involves a defect in tolerance induction [24]. A number of potential mechanisms link breastfeeding to tolerance induction, including the presence of antigens and tolerogenic factors in breast milk, as well as effects on gut microbiota and permeability [24]. However the evidence for a protective role of breastfeeding is mixed [25,26], and in the PROBIT trial, whilst there was a marked difference in exclusive breastfeeding at 3 months (44.3% in intervention *vs.* 6.4% in control group) there were no differences in risk of asthma or allergy in the children followed up at 6.5 years [27]. One possibility is that misclassification of infant feeding exposures is particularly problematic in studies of atopic disease. Hypersensitivity reactions may not show a dose-dependence, and they may be affected by frequency and timing of exposures [27]—both of which may be poorly characterised. For infants who are not breastfed, formula milks containing hydrolysed protein and prebiotics have been recommended for the prevention of atopic outcomes. There is currently little evidence of benefit of feeding hydrolysed protein formulas, when compared to exclusive breast feeding [28], although limited evidence of benefit for high-risk infants, when compared to a cow’s milk formula. There is currently insufficient evidence to assess the role of prebiotic supplementation of infant formula for prevention of allergic disease [29].

In terms of other aspects of infant feeding, very early introduction of solid foods may increase the risk of eczema, but currently there are few data to support an association with other allergic conditions [26,30]. In a systematic review of the relationship between early introduction of solid foods and development of allergic disease, Tarini and colleagues highlight the difficulties of exposure ascertainment, and the lack of systematic definition of the quantity and quality of solid foods introduced [30]. The age at which solid foods should be introduced and the need for delayed introduction of allergenic foods remains controversial and further studies are needed [26,30,31].

### 3.3. Intelligence and Cognitive Development

Brain growth is rapid in the first year of life, and slow growth in infancy predicts poor cognitive performance in later life and lower educational attainment [32,33,34]. This has led to considerable interest in the influence of infant feeding on cognitive development, in particular breastfeeding, which has been linked to higher intelligence in a number of studies [35]. Breast milk may support neurocognitive development by providing long-chain polyunsaturated fatty acids, which are found in high concentrations in the brain, and accumulate during the period of rapid growth [35]. Other bioactive constituents in breast milk, such as nucleotides, may also have important roles [36], although we currently know little about these. 

In high-income countries, children who were breastfed in infancy are commonly observed to have better performance in tests of cognitive function. However the decision to breastfeed and duration of breastfeeding are often strongly linked to family background, particularly to maternal intelligence, which may be overlooked as a confounder [37]. In a meta-analysis of observational studies in 2006, Der and colleagues showed that when maternal intelligence is accounted for, the differences in children’s performance are no longer evident, suggesting that apparent benefits of breastfeeding could be due to effects of residual confounding [37]. In 2008, data from the PROBIT trial were published, which showed that the intervention was associated with a difference of 5.9 points for full-scale IQ when the children were assessed at age 6.5 years [38]. Although the findings have been debated [39], the experimental design of the study means that the data lend strong support to benefits of breastfeeding for cognitive development. Further evidence of a causal link between breastfeeding and intelligence has come from the recent comparison of UK children in the ALSPAC cohort with Brazilian children in Pelotas [8]. In both cohorts, despite differences in the effects of social background on breastfeeding, that were marked in the UK but not observed in Pelotas, there was a positive association between breastfeeding duration and IQ. Brion and colleagues comment that adjustment for confounders attenuated the association in both cohorts, thus residual confounding is still a possibility. They recommend that further large studies are needed from populations where confounding structures differ [8].

There is little evidence of links between other aspects of weaning practice and cognitive development. In one study, children whose weaning diet was characterised by higher intakes of fruit, vegetables and home-prepared foods had higher scores on tests of full-scale and verbal intelligence at age 4 years. This association was independent of a range of confounding factors, including maternal intelligence and breastfeeding duration [40]. Although weaning practice is also socially patterned [10], and there may be effects of residual confounding, these data suggest that the quality of the weaning diet could affect brain development when growth velocity is high. Alternatively, as dietary patterns “track” in childhood [41], this association may reflect continued exposure to diets that provide an optimal supply of micronutrients to support cognitive development. Further work is needed to determine the influence of qualitative differences in the weaning diet on later cognitive function. 

### 3.4. Body Composition and Obesity

As a number of epidemiological studies have shown a lower risk of obesity in children and adults who were breastfed, infancy has become a focus of public health interest as a critical period that could be targeted for obesity prevention [42,43]. There are a number of reasons why being breastfed might be protective. One possibility is that as breastfed babies control the amount of milk they consume, they learn effective self-regulation of energy intake, which remains with them in later life [44,45]. Secondly, in comparison with formula-fed infants, breastfed infants gain weight more slowly in infancy [46], and slow infant weight gain is associated with a lower risk of later obesity [47]. Although this might appear the simplest explanation, it may not be straightforward, as one study identified the critical period associated with later adiposity as early infancy [48], before differences in weight gain between breastfed and formula-fed infants are evident [46]. Another possibility is that there are bioactive components in breast milk that have long-term programmed effects on function—for example early exposure to leptin and adiponectin in breast milk could be involved in setting endocrine responses to feeding and appetite regulation [49]. Little is currently known about such programmed effects, but the trial finding that human milk intake in pre-term infants is associated with lower leptin levels relative to fat mass in adolescence may be of importance [50]. 

Early systematic reviews that linked breastfeeding to later body composition in high-income populations showed that evidence of a protective effect on obesity was reasonably consistent across studies [51,52]; in a meta-analysis, Harder and colleagues estimated that risk of becoming overweight in later life was reduced by 4% for each additional month of breastfeeding [53]. However, effects on mean BMI are small [54], and recent analyses of observational data from low and middle-income countries have not confirmed the inverse association. In data pooled from five prospective birth cohort studies of more than 10,000 young adults, there was no association between being breastfed and later adiposity, nor was there any evidence of benefits of a longer duration of breastfeeding [7]. This difference in findings was also evident in a comparison of UK children in the ALSPAC study with a contemporary cohort of children born in Pelotas, Brazil, where there was little social patterning of breastfeeding [8]. Longer duration of breastfeeding was associated with lower BMI in the children studied in the UK, but this association was not found in the Brazilian children. These findings are in accord with the PROBIT trial which showed no difference in BMI in the children in the intervention and control groups at 6.5 years [55], suggesting that residual confounding could be the explanation for the inverse associations previously seen in observational studies. 

However, further data are needed. Firstly, BMI may be too crude a measure of differences in body composition, particularly in childhood [56]. In prospective studies in which fat mass has been measured directly using DXA, differences in adiposity in children have been found in relation to duration of breastfeeding, even after adjustment for a range of known determinants of adiposity and other confounding factors [57,58]. Importantly, this association may not be evident when using BMI as a marker of fat mass [58]. Secondly, studies of siblings have shown differences in adiposity in relation to breastfeeding, which cannot be explained by differences in social background or lifestyle [59,60]. In this respect, future studies of adiposity using direct measurement techniques will be important, particularly in populations where influences on infant feeding practice and confounding structures differ.

Less is known about other aspects of infant feeding in relation to later obesity. Although early introduction of solid foods has been linked to obesity in some studies [7,21], this is not a consistent finding [61]. Recent data from the Project Viva cohort study in the US suggest that some of the inconsistency may be explained by differences between breastfed and formula-fed infants [62]. Among formula-fed infants, introduction of solid foods before 4 months was associated with a six-fold increase in risk of obesity at 3 years (adjusted odds ratio 6.3, 95% CI 2.3 to 6.9), whereas the timing of solid food introduction was not associated with later obesity risk among the breastfed infants. The authors suggest that breastfeeding may promote self-regulation of energy intake, leading to a reduction in milk consumption when these infants are given solid foods. In contrast, formula-fed infants increase their energy intake [62]. These findings highlight the need to consider the combined influences of milk feeding and the nature and timing of the weaning diet when evaluating the role of infant nutrition in long-term health outcomes.

### 3.5. Cardiovascular Disease

Evidence collated over the past decade has enabled an evaluation of the links between variations in infant feeding and cardiovascular disease and its risk factors in adult life [2]. The primary sources of data are observational, from historical cohorts that had documented reports of infant feeding as well as adult data collected at follow-up. The principal exposures that have been considered are duration and exclusivity of breastfeeding, plus the comparison of breastfed individuals with those fed breast milk substitutes in infancy. There is little information on other aspects of infant feeding in relation to later cardiovascular disease.

#### 3.5.1. Blood Cholesterol

As breast milk contains cholesterol, there are marked differences between breast and formula-fed infants in their exposure to dietary cholesterol in early infancy, and studies of breastfed infants show that they have higher total blood cholesterol concentrations when compared with other infants [63]. It has been proposed that the early exposure of breastfed infants to dietary cholesterol could result in programmed effects on endogenous cholesterol synthesis, leading to differences in regulation, that are evident later in life. Pooled analyses of observational data that link type of milk feeding to total cholesterol concentrations in adult life support this proposition. Although children who were breastfed or formula-fed in infancy do not show differences, in adult life, breastfed individuals have lower blood cholesterol concentrations (−0.18 mmol/L, 95% CI −0.30, −0.06 mmol/L) [63]. A further analysis by Owen and colleagues in 2008 showed that the protective effect of breastfeeding was robust to adjustment for a range of adult confounders, which included smoking and BMI [64]. Although the differences in cholesterol were small, and there were insufficient data to examine the dose-effects of duration of breastfeeding, the effects were more marked among adults who had been exclusively breastfed, who were likely to have been breastfed for longer. The age-dependence of the protective effect [63], suggests that the children in the intervention studies [3,4] may currently be too young to determine whether there are differences in total cholesterol resulting from differences in breast milk exposure in infancy. In a follow-up study of the preterm infants randomised to breast milk or pre-term formula, differences in total cholesterol between the groups at age 13–16 years were of borderline significance [65], but there were differences in other lipoproteins, including a lower ratio of LDL to HDL cholesterol in the breast milk group.

#### 3.5.2. Blood Pressure

Meta-analyses of observational data have shown that breastfeeding is associated with small reductions in systolic blood pressure in later life [66,67]. A smaller difference in diastolic blood pressure in relation to breastfeeding is evident in some studies, but this is a less consistent finding. The mechanisms that link breastfeeding to later blood pressure are unknown, but may be due to programmed effects of bioactive or other dietary constituents in breast milk that are absent from formula milk [68]. In the meta-analysis by Martin and colleagues in 2005, the difference in SBP attributed to being breastfed was −1.4 mmHg (95% CI −2.2, −0.6). If causal, this could confer important benefits at a population level [67]. The follow-up study of pre-term infants at age 13–16 years, randomized to breast or pre-term milk in infancy, provide further support for the findings of the meta-analyses; in a non-randomized analysis, the proportion of intake as human milk was inversely related to later mean arterial blood pressure [68]. 

However, these findings differ from those of the PROBIT trial [55]. Despite a much higher prevalence and duration of exclusive breastfeeding resulting from the intervention in infancy, there were no differences in systolic or diastolic blood pressure between the children in the intervention and control groups, when they were followed up at 6.5 years. Furthermore, in the recent comparison of children in the ALSPAC and Pelotas cohorts, whilst an inverse association was found between breastfeeding duration and SBP in the UK children that was independent of confounders, this was not evident in children from Pelotas [8]. Fall and colleagues also show weak and inconsistent findings between duration of breastfeeding and SBP or risk of hypertension, in their analysis of data from young adults in low and middle-income populations [7]. These recent findings, together with heterogeneity across studies observed in the meta-analyses [66,67] raise the possibility that residual confounding could explain the inverse association observed between breastfeeding and blood pressure. However, further data are needed, particularly from populations where influences on feeding practice differ, and to address effects of exclusive breastfeeding [7].

#### 3.5.3. Blood Glucose and Type 2 Diabetes

A systematic review of observational studies by Owen and colleagues in 2006 showed an association between breastfeeding and later risk of type 2 diabetes [69]. Based on studies mainly conducted in high income countries, being breastfed was associated with a substantial reduction in risk (OR 0.61, 95% CI 0.44, 0.85). This effect was reasonably consistent across studies, and among non-diabetic participants, being breastfed was associated with slightly lower fasting insulin concentrations in later life [69]. A protective effect of breastfeeding may be explained by the bioactive components of breastmilk [70] or the content of LCPUFA, which in the past were absent from formula milks. For example, breastfeeding is associated with greater LCPUFA levels in skeletal muscle membranes, which in turn have been shown to be associated with lower fasting plasma glucose [70,71]. 

But the recent analysis of data from birth cohorts of young adults in low and middle-income countries do not confirm the findings of the meta-analysis, as there was no association between being breastfed, or duration of breastfeeding on plasma glucose concentration or risk of type 2 diabetes [7]. There are differences in breastfeeding initiation rates between the two sets of analyses, as breastfeeding was almost universal in the low and middle-income countries, which may be important, but the disparate findings also raise the possibility of residual confounding as an explanation for the association previously observed. In contrast, new data from a follow-up study of 9-year-old children in an Indian cohort show that longer duration of breastfeeding in infancy was associated with lower 2 h plasma glucose concentrations in a glucose tolerance test, indicating protective effects of breastfeeding that are consistent with earlier studies [72]. Further data from contemporary populations in low and middle-income countries are clearly needed, particularly among those undergoing rapid transition to urbanised and Western lifestyles.

#### 3.5.4. Cardiovascular Disease

Although breastfeeding is associated with reductions in a number of risk factors for cardiovascular disease, consistent effects of breastfeeding on CVD have not been found. In a systematic review and meta-analysis of four observational studies from the UK and US in 2004, Martin and colleagues showed there was no association between breastfeeding and mortality from cardiovascular disease [73]. Following this review, a follow-up study of 87,252 women in the Nurses’ Health Study, found small reductions in risk among those who had been breastfed (adjusted hazard ratio 0.91 (95% CI 0.83–1.01) for any cardiovascular event) [74]. It is not clear why these studies differ, or the reasons for differing associations when compared with cardiovascular risk factors, although there may be particular challenges in defining nutritional exposures in infancy in historical cohorts. At present there are limited data to be able to address this.

### 3.6. Dietary Preference and Food Choice

The epidemiological associations that link variation in infant feeding to adult health suggest that there are permanent programmed effects on function that result from differences in dietary exposures in early postnatal life. However, an alternative explanation is that differences in exposure to breast milk, and variations in the nature and timing of weaning, have long-term effects on food choice and dietary behaviour [64]. There is some evidence that breastfeeding affects acquisition of taste preferences in infancy [75]—and it is possible that breastfed infants simply have “healthier” dietary patterns in later life. Although this is challenging to address, as social patterning effects are evident both in relation to breastfeeding behaviour and adult diet, there is growing experimental evidence that early dietary exposures can affect taste preference. For example, infants fed bitter-tasting protein hydrolysate formula milks show a greater preference for foods with the same sensory attributes in later childhood [76,77,78]. Breastfeeding exposes infants to volatile flavour compounds from the maternal diet, and in comparison with other infants, these exposures are much more variable. This may have a significant impact on flavour learning in infancy [76,79], and experimental evidence suggests later acceptance of foods is greater among children who were breastfed [79,80]. Consistent with this finding, greater exposure to breast milk has been linked to healthier dietary habits in adult life in two recent studies [81,82], suggesting that that such effects on taste preference and/or food acceptance could be important for life-long food habits, and may contribute to the beneficial effects of breastfeeding observed in relation to adult health outcomes. Further prospective studies are needed, particularly from populations where social patterning effects on infant feeding practice are less evident.

## 4. Conclusion

There is a growing recognition of the need for a lifecourse approach to understanding the aetiology of adult disease, and there is now a significant evidence base that links patterns of infant feeding to differences in health outcomes, both in the short and longer term. In particular, observational data show that being breastfed is associated with lower rates of infection in infancy, and with reductions in blood pressure, cholesterol, and lowered risks of obesity and diabetes in adult life. Although the effects on cardiovascular risk factors are modest, breastfeeding rates are suboptimal in many countries [12,16], and strategies to promote breastfeeding could therefore confer important benefits for cardiovascular health at a population level. Given the difficulties in assessing exposure to breast milk and other associated aspects of infant feeding it is likely that there is considerable misclassification of individuals, and the identified associations may have been underestimated. There are currently few data to show whether other aspects of infant feeding practice, such as age at introduction of solid foods, or qualitative differences in the weaning diet are determinants of later outcomes.

A significant limitation of the existing evidence is that it has mainly come from studies in high-income countries, where there are clear social gradients in infant feeding practice, particularly in breastfeeding duration. Despite appropriate statistical adjustments, the possibility of residual confounding remains. New data from low and middle-income populations of children and young adults, where the determinants of infant feeding behaviour differ, suggest that for blood pressure, BMI and risk of diabetes, confounding may be an explanation for the associations seen in earlier analyses. In comparison with high-income countries, low and middle-income countries also differ in prevalence of breastfeeding and in their patterns of adult disease, and many are undergoing rapid transition to Western lifestyles [83]. It will be important to continue to follow up these and other comparable study populations in the future, to be able to assess the effects of transition, and to determine associations with morbidity and mortality at older ages. 

Whilst further data are needed, misclassification of nutritional exposure in infancy remains a key challenge. Future progress will depend on new studies that provide detailed prospective data on duration and exclusivity of breastfeeding, together with appropriate characterisation of the weaning diet. 
